# Robust Real-Time Music Transcription with a Compositional Hierarchical Model

**DOI:** 10.1371/journal.pone.0169411

**Published:** 2017-01-03

**Authors:** Matevž Pesek, Aleš Leonardis, Matija Marolt

**Affiliations:** 1 University of Ljubljana, Faculty of Computer and Information Science, Laboratory for computer graphics and multimedia, Ljubljana, Slovenia; 2 University of Birmingham, School of Computer Science, Centre for Computational Neuroscience and Cognitive Robotics, Birmingham, United Kingdom of Great Britain and Northern Ireland; Georgia Institute of Technology, UNITED STATES

## Abstract

The paper presents a new compositional hierarchical model for robust music transcription. Its main features are unsupervised learning of a hierarchical representation of input data, transparency, which enables insights into the learned representation, as well as robustness and speed which make it suitable for real-world and real-time use. The model consists of multiple layers, each composed of a number of parts. The hierarchical nature of the model corresponds well to hierarchical structures in music. The parts in lower layers correspond to low-level concepts (e.g. tone partials), while the parts in higher layers combine lower-level representations into more complex concepts (tones, chords). The layers are learned in an unsupervised manner from music signals. Parts in each layer are compositions of parts from previous layers based on statistical co-occurrences as the driving force of the learning process. In the paper, we present the model’s structure and compare it to other hierarchical approaches in the field of music information retrieval. We evaluate the model’s performance for the multiple fundamental frequency estimation. Finally, we elaborate on extensions of the model towards other music information retrieval tasks.

## Introduction

Music information retrieval (MIR) deals with extraction of semantic descriptions from music in its various forms. As in many related areas, a significant increase in algorithm accuracy and efficiency has been achieved in recent years for tasks such as melody estimation [1, 2], chord estimation [3–5], beat tracking [6, 7], mood [8] and genre estimation [9, 10], and pattern analysis [11–13].

Recently, parallel to other areas, deep learning has been successfully introduced to the MIR [14, 15]. A deep learning algorithm constructs multiple levels of data abstraction (a hierarchy of features) in order to model high-level representations present in the observed data [16]. Several deep learning models have been applied to different MIR tasks, such as deep neural networks, convolutional neural networks (CNNs) and deep belief networks (DBNs). One of the first uses of deep architectures for analyzing audio signals was presented by Lee [17], who applied convolutional DBNs for speaker identification. Later, Hamel and Eck [18], evaluated DBNs for genre recognition using a five-layer DBN with three hidden layers for feature extraction. Since then, deep architectures achieved promising results on a variety of tasks: Schmidt and Kim [19] used a five-layer DBN for extraction of emotion-based acoustic features, Pikrakis [20] showed that DBNs can be used for rhythm genre discrimination, conditional DBNs were used by Battenberg and Wessel [21] for drum pattern analysis, while Schmidt [22] showed that DBNs can be trained to understand rhythm and melody. Other architectures have also been used, for example recurrent neural networks for audio chord estimation [5, 23] and convolutional neural networks for key detection [24] and onset detection [25, 26].

The goal of music transcription is to estimate a music score (notes played) from an audio signal. Its essential part is the multiple fundamental frequency estimation, where the goal is to estimate all the fundamental frequencies (corresponding to pitches) in individual time-frames of a music signal. As an important MIR goal, transcription has been researched since the early 1970s and a variety of approaches have been developed [27–30]. Some approaches use note hypothesis evaluation based on the signal spectrum [31, 32], while others [33–35] model the audio signal as a composition of sources. Several approaches are tuned to the transcription of specific instruments [36–39] or focus on transcribing instrument-specific symbolic data [40].

Several neural-network-based deep approaches were also presented for music transcription [41–44]. Bock and Schedl [41] used a recurrent neural network model for a piano transcription, while Nam et al. [42] combined deep belief networks with support vector machines and a hidden Markov model for the same task. Rigaud and Radenen [44] proposed a combination of two deep neural networks for transcription of singing voice.

However, music transcription approaches are rarely evaluated on the real-world recordings, which may not have been recorded in ideal studio environments or with professional performers. This is in large part due to the lack of diverse annotated datasets currently available—most datasets consist mainly of the synthesized recordings, which are easily obtainable, and contain only a small number of annotated real recordings. Consequently, the robustness of the algorithms may suffer, as they may overfit the small datasets and the instrument timbres, which leads to poor performance on diverse materials and in the presence of noise.

This paper introduces a novel compositional hierarchical model for the multiple fundamental frequency estimation (MFFE). The proposed model can be regarded as a novel deep architecture with unsupervised learning and a transparent structure, which allows for representation and interpretation of the signal’s content on different levels of complexity. The model’s main feature is the relativity of learned concepts, which enables construction of compact and robust models. The main contribution of this paper is a model which can perform robustly on datasets that vary in audio and source quality, with real-time computation and affordable spatial requirements. This makes the model useful for a wide range of applications and with music recordings of varying quality.

The presented model is an extension of the model first introduced in [45] for three different MIR tasks: chord estimation, mood estimation and MFFE. Its structure and learning algorithm are improved, resulting in higher MFFE accuracy. Additionally, the experimental part is significantly extended in this paper using four datasets for a cross-dataset evaluation. We also adapted the model for melodic pattern extraction in the symbolic domain [46], where we evaluated it on the JKU PDD dataset. For each musical piece, the model was built independently and inferred with the same piece. Patterns were represented by activations of parts on the top layers.

The paper is structured as follows: the proposed model is described in the first Section. The evaluation of the model is provided in the second Section, followed by discussion. The last Section concludes the paper and gives ideas for future work.

## Compositional hierarchical model for MIR

The main principle of compositional hierarchical models lies in the hierarchical nature of our perception of the world. Just like our visual system can discern complex forms by combining basic elements like edges, lines, contrasts and colors into increasingly more complex percepts, so can our auditory system group frequency components into auditory events, multiple tonal events into harmonies, their time evolution into melodies and harmonic progressions.

Hierarchical music representations are intuitive when considering the spectral and temporal structures in music. The generative theory of tonal music [47] may well be the first examples of hierarchical music modeling in musicology. Although the model itself mostly relies on expert rules, the hierarchical structuring is a good fit, since it is based on patterns of human perception and cognitive processes. Other attempts [48, 49] have been made to empirically evaluate such hierarchical representations produced by human cognitive processes. The approaches based on temporal hierarchical structures [50] have been presented, taking human short-term memory into consideration, while defining a rule-based model for auditory processing. Hierarchical models also abound in analysis of music perception from the point of view of computational biology and neuroscience [51–54].

We propose a compositional hierarchical model designed specifically for music signal processing. The model can learn a hierarchical representation of audio signals in an unsupervised manner, starting from signal components on the lowest layer, up to individual music events on the highest layers.

The structure of our model is inspired by the research in the field of computer vision, specifically the *learned Hierarchy of Parts* (lHoP) model presented by Leonardis and Fidler [55, 56]. Their model represents objects in images in a hierarchical manner, structured in layers, from simple to complex image parts. The model is learned from the statistics of natural images and can be employed as a robust statistical engine for object categorization and other computer vision tasks.

We show that a similar approach can also be used for music representation and analysis. Our model is built on the assumption that a complex signal can be decomposed into a hierarchy of building blocks—*parts*. The parts exist at various levels of granularity and represent sets of entities describing the signal. According to their complexity, parts can be structured across layers from the less to the more complex. The parts on higher layers are expressed as compositions of parts on lower layers, analogous to the fact that a chord is composed of several pitches, and each pitch of several harmonic partials. A part can therefore describe individual frequencies in a signal, their combinations, as well as pitches, chords and temporal patterns, such as chord progressions. The entire structure is *transparent*, so that the role of each part can be observed and interpreted.

The presented model differs from the aforementioned lHoP model in its concept. While it shares the inspiration for its hierarchical composition of structures and statistical learning, the CHM was developed from scratch with focus on MIR tasks. The input to the CHM is a spectral audio representation, which significantly influences its structure. Consequently, the mechanisms for activations, part compositions and layers were redefined to meet the specifics of such representation. Inhibition and hallucination mechanisms, also inspired by the lHoP model, were newly defined according to the new model structure. Additionally, an automatic gain control mechanism that incorporates time dimension into CHM processing was newly introduced specifically for this model.

### Model structure

**Fig 1:**
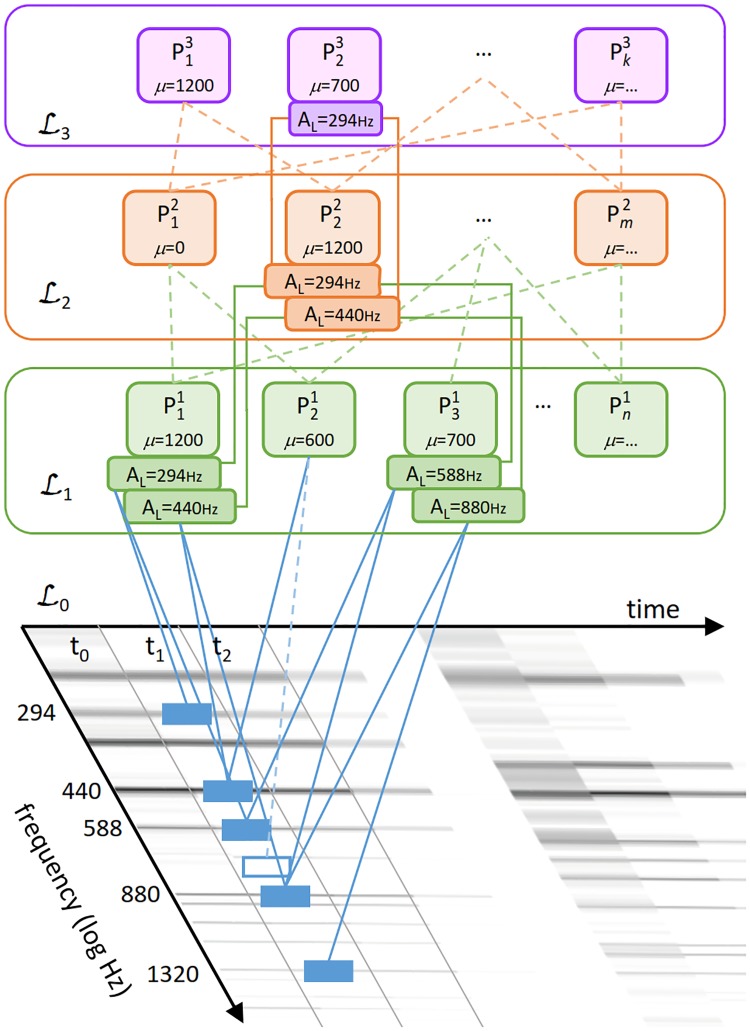
The compositional hierarchical model. The input layer corresponds to the signal components in the time-frequency representation. The parts on higher layers are compositions of the lower-layer parts (depicted as connections between parts, parameter *μ* is given in cents). A part may be contained in several compositions, e.g. $P_{1}^{1}$ is a part of compositions $P_{1}^{2}$, $P_{2}^{2}$ and $P_{m}^{2}$. Active parts have activation locations displayed underneath, a part can have several activations on different locations. The entire structure is transparent, thus we can discern that the activation of $P_{2}^{2}$ at 294*Hz* represents a tone (harmonic series) starting at 294 Hz by observing the subtree leading from the activation to $L_{0}$.

The compositional hierarchical model consists of the input layer $L_{0}$ and several compositional layers $\left\{L_{1},\ldots ,L_{N}\right\}$. Each compositional layer $L_{n}$ contains a set of parts $\left\{P_{1}^{n},\ldots ,P_{M}^{n}\right\}$, where a part is a composition of parts from $L_{n-1}$ and may itself be part of any number of compositions on $L_{n+1}$. Thus, the compositional model forms a hierarchy of parts, as may be observed in Fig 1, where connections between the parts represent the structure of compositions.

#### Compositional layers

Layers $\left\{L_{1},\ldots ,L_{N}\right\}$ contain parts which are compositions of parts from lower layers. Formally, we define the composition $P_{i}^{n}$ as:

$$\begin{array}{c} P_{i}^{n}=\left\{P_{k_{0}}^{n-1},{\left\{P_{k_{j}}^{n-1},\left(\mu_{j},\sigma_{j}\right)\right\}}_{j=1}^{K-1}\right\}. \end{array}$$ (1)

$P_{i}^{n}$ is a composition of *K* parts from layer $L_{n-1}$—*subparts*. The composition is governed by the parameters *μ*_1, …, *K* − 1_ and *σ*_1, …, *K* − 1_ which model relations between subparts. These relations are *relative*, meaning that the compositions are defined by the relative distances (*offsets*) between the subpart $P_{k_{0}}^{n-1}$ and the subparts $P_{k_{1}}^{n-1},\ldots ,P_{k_{K-1}}^{n-1}$. The offsets are encoded by parameters *μ*_1, …, *K* − 1_ and *σ*_1, …, *K* − 1_ and always defined relative to $P_{k_{0}}^{n-1}$ which we denote as the composition’s *central* part. For example, $P_{2}^{2}$ in Fig 1 is defined as:

$$\begin{array}{c} P_{2}^{2}=\left\{P_{1}^{1},\left\{P_{3}^{1},\left(1200,25\right)\right\}\right\}, \end{array}$$ (2)

where *μ* and *σ* are given in cents. It represents a composition of $P_{1}^{1}$ with $P_{3}^{1}$ spaced approximately 1200 cents (one octave) apart, where *σ* governs the allowed deviation from this value. Since all relationships in the model are relatively encoded, rather than encoding specific instances of a music concept (e.g. the tone A5), our model learns generalized concepts (e.g. a tone is a set of frequency components at some relative positions). The benefits of such relative encoding are discussed in the *Relativity and shareability of parts* Section. All compositions and their parameters are learnt in an unsupervised manner, as explained in the *Learning* Section.

The mapping from relatively defined to absolutely positioned concepts (e.g. a generalized tone concept to the tone A5) is performed during an *inference* on an input audio signal, by calculating part *activations* upwards through all the layers (see *Inference* Section).

A part *activation* indicates that the concept it represents was found in the input signal. An activation has two components: a *location*, which maps the part onto the frequency axis, thus making it absolute, and a *magnitude*, representing its strength. A part can activate only if all of its subparts are activated with magnitude greater than zero (this constraint can be relaxed as described in the *Inference* Section). Due to the relative encoding of the concepts in the model, a part can simultaneously activate at multiple locations, indicating that the concept it represents was found at several locations in the input signal.

The activation location of part $P_{i}^{n}$ at time *t* is defined as:

$$\begin{array}{c} A_{L}^{\left(t\right)}\left(P_{i}^{n}\right)=A_{L}^{\left(t\right)}\left(P_{k_{0}}^{n-1}\right). \end{array}$$ (3)

Thus, central parts of compositions propagate their locations upwards through the hierarchy. With respect to the example in Eq 2, when $P_{1}^{1}$ is activated at 440*Hz*, $P_{3}^{1}$ at 880*Hz*, and $P_{2}^{2}$ is activated at 440Hz. Such propagation of the locations through the central parts represents a very useful indexing mechanism, which enables an efficient top-down analysis of part activations from the upper to the lower layers, adding to the transparency of the model.

The activation magnitude is defined as a weighted sum of subpart magnitudes:

$$\begin{array}{c} \begin{array}{cc} & w_{j}=\left\{\begin{array}{cc} 1: & j=0\\ N(A_{L}^{\left(t\right)}\left(P_{k_{0}}^{n-1}\right)-A_{L}^{\left(t\right)}\left(P_{k_{j}}^{n-1}\right),\mu_{j},\sigma_{j}): & j>0 \end{array}\right.\\ & A_{M}^{\left(t\right)}\left(P_{i}^{n}\right)=\tanh\left(\frac{1}{K}\sum_{j=0}^{K-1}w_{j}A_{M}^{\left(t\right)}\left(P_{k_{j}}^{n-1}\right)\right) \end{array}, \end{array}$$ (4)

where the weights *w*_*i*_ are defined by the match between the locations of the subpart activations and the composition parameters *μ* and *σ*.

#### Input layer

The input layer $L_{0}$ models a time-frequency representation of the input signal *X*. It consists of the single atomic part $P_{1}^{0}$, which is activated at locations of all the frequency components in the signal at a given time-frame *t*. Thus, for any frequency bin *k*, $P_{1}^{0}$ is activated as:

$$\begin{array}{c} \begin{array}{l} A_{L}^{\left(t\right)}\left(P_{1}^{0}\right)=f\left(k\right)\\ A_{M}^{\left(t\right)}\left(P_{1}^{0}\right)=\left|X_{t}\left(k\right)\right|, \end{array} \end{array}$$ (5)

where *f*(*k*) represents the frequency of the frequency bin *k* and |*X*_*t*_(*k*)| its magnitude.

### Relativity and shareability of parts

The proposed model has two important features that set it apart from similar architectures.

The *relativity* of parts enables a single part to represent an abstract high-level concept regardless of its location in the input signal. Relative perception naturally occurs in human learning process. It is an important part of the abstraction of the object of interest, and enables the formation of a complete percept, regardless of its environment. It minimizes the amount of memory needed to store the learned concepts and enables their robust identification in previously unobserved sensory inputs, such as within noisy audio signals and in the presence of non-musical events.

Relativity is inherent in our model and can be observed in the definitions of part composition and activation (Eqs 1 and 4). Although the parts are relative and only represent abstract concepts with no direct absolute representation (e.g. the model cannot encode the pitch G5 explicitly, but only the concept of pitch), the part’s activation at a given location indicates where and when a given concept appears in the signal. Since this can occur at several locations, a part can have multiple activations at different locations. This is also shown in Fig 1, where $P_{1}^{1}$, $P_{3}^{1}$ and $P_{2}^{2}$ have two activations each, meaning that the concepts they represent are present at several locations in the signal.

The relative nature of parts that enables the representation of concepts regardless of their location also enables efficient *shareability* of the parts. A single part on the layer $L_{n-1}$ may be a part of several compositions on the layer $L_{n}$. Consequently, any two or more $L_{n-1}$ parts may form a number of different $L_{n}$ compositions at different offsets. Thus, they may be combined into several more complex abstractions, themselves relative.

The consequence of relativity and shareability is that the model can very efficiently encode complex concepts. As an example: a part representing the concept of pitch may be shared by several compositions on a higher level that encode different intervals. This encoding is general, compact and efficient if we consider the alternative of encoding all the intervals in an absolute manner. This is also evident in the evaluation of the proposed model (see *Evaluation* Section), where a learned hierarchy with a small number of compositions is shown to be robust and to generalize well in modeling musical events in audio signals, which differ from the ones used for training in quality, the amount of noise and the number and the type of sources present in the signal.

### Learning

The model is constructed layer-by-layer with unsupervised learning on a set of input signals, starting with $L_{1}$. We view the learning as an optimization problem, where we aim to find a minimal set of compositions for the learned layer, which will explain the maximal amount of information present in the input data. The learning process is driven by the statistics of part activations which capture regularities in the input data.

To formalize the problem, we first define the *coverage* of a part’s activation at the time *t* as a set of $L_{0}$ activations (spectral components) which have caused the activation. This set can be obtained efficiently by observing the tree formed by the activated subparts through indexing encoded in the locations of their central parts down to the layer $L_{0}$ as:

$$\begin{array}{c} \begin{array}{c} A_{C}^{\left(t\right)}\left(P_{i}^{n}\right)=\overset{K-1}{\underset{j=0}{\cup }}A_{C}^{\left(t\right)}\left(P_{k_{j}}^{n-1}\right)\\ A_{C}^{\left(t\right)}\left(P_{1}^{0}\right)=\{k:f\left(k\right)\in A_{L}^{\left(t\right)}\left(P_{1}^{0}\right)\}. \end{array} \end{array}$$ (6)

The coverage of the entire layer $L_{n}$ is the set of spectral components in the input data, which all the parts in the layer cover:

$$\begin{array}{c} A_{C}^{\left(t\right)}\left(L_{n}\right)=\underset{p\in L_{n}}{\cup }A_{C}^{\left(t\right)}\left(p\right) \end{array}$$ (7)

The goal of learning a new layer $L_{n}$ is to minimize the amount of uncovered information in the input data and, on the other hand, to limit the number of parts added to the layer, which can be expressed as:

$$\begin{array}{c} min(\sum_{t}\sum_{k\notin A_{C}^{\left(t\right)}\left(L_{n}\right)}{\left|X_{t}\left(k\right)\right|}^{2}+\lambda \left|L_{n}\right|), \end{array}$$ (8)

where *λ* is a regularization factor which balances between the number of parts and the adequacy of the coverage.

The problem of finding an optimal coverage is a special case of the well-known set cover problem, which is NP-complete. We therefore approximate the solution by using a greedy algorithm, which incrementally adds compositions to the new layer. With each iteration the algorithm chooses a composition that covers the largest amount of uncovered data. The entire learning algorithm is composed of two steps: finding new candidate compositions and adding compositions to a new layer.

#### Finding candidate compositions

**Fig 2:**
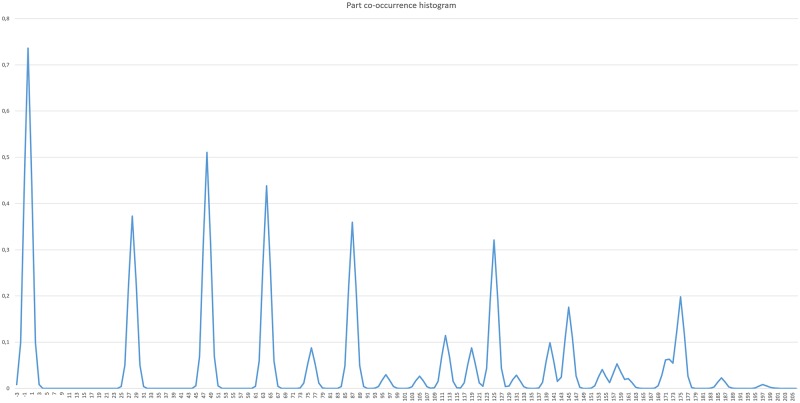
Co-occurrence histogram for an $L_{2}$ part. The normalized co-occurrence histogram represents the distribution of distances (offsets) of $L_{2}$ subparts that activate simultaneously. The distances are shown relative to a chosen central $L_{2}$ part.

When learning the layer $L_{n}$, we first need to form a set of new compositions, which will be considered for inclusion in the new layer. We perform inference on the training set up to the layer $L_{n-1}$, and then observe the co-occurrences of $L_{n-1}$ part activations over the entire training set. The co-occurrences provide information on the parts, which frequently activate simultaneously and are thus believed to form a common concept. We calculate the histograms of co-occurring activations according to distances between activation locations. New compositions are formed from parts where the number of co-occurrences exceeds a learning threshold *τ*_*L*_. The composition parameters *μ* and *σ* are estimated from the corresponding histogram (Fig 2) and each new composition is added to the set of candidate compositions C.

#### Selecting compositions

**Fig 3:**
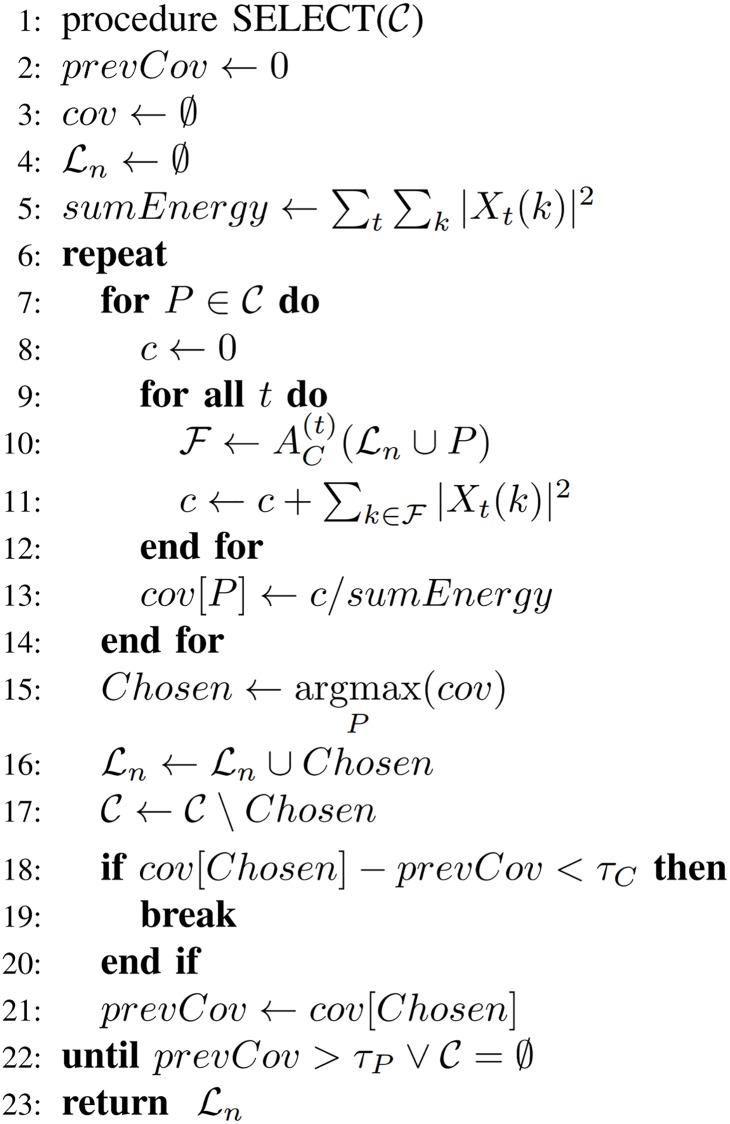
Greedy algorithm for the selection of compositions from the candidate set C. Compositions that add the most to the coverage of information in the learning set are prioritized.

Due to the NP-completeness of the set cover problem, we use a greedy approach to select a subset of compositions from the set C, which leaves a minimal amount of information in the training set uncovered (according to Eq 8). In each iteration, a composition from C, which contributes the most to the coverage of the training set, is selected and added to the new layer. This ensures that only compositions which provide enough new information with regard to the currently selected set will be added. The selection is stopped when either: a sufficient percentage of information in the learning set is covered (according to the threshold *τ*_*P*_), or no part from the candidate set adds enough to the coverage of information (according to *τ*_*C*_). The algorithm in Fig 3 outlines the described approach.

The learning proceeds layer-by-layer, starting at $L_{1}$, until the complexity of the layer parts achieves the desired complexity of modeled musical events, depending on the underlying problem. The chosen values of thresholds and their impact on training are described in the *Evaluation* Section.

### Inference

Inference is the process of calculating part activations on an input signal according to Eqs 3 and 4. Inference is calculated bottom-up and layer-by-layer, whereby the time-frequency representation of the input signal serves as the input of the layer $L_{0}$. The observed locations and magnitudes of activations yield insight into the analyzed signal through the concepts that the activated parts represent and can be used as features for further processing.

In this Section, we describe three additional mechanisms that can be used during an inference to increase the predictive power and robustness of the model: *hallucination*, *inhibition* and *automatic gain control*.

#### Hallucination

When calculating activations, the default model behavior is very conservative—a part is activated only if all of its subparts are activated (*Model structure* Section). *Hallucination* relaxes this condition and enables the model to produce activations even in the case of incomplete (missing, masked or damaged) input. The model generates activations of parts, which most fittingly cover the information, present in the input signal, where fragments, which are not present, are “hallucinated”. The missing information is thus extrapolated from the knowledge acquired during learning, encoded into the model structure.

Hallucination changes the conditions under which a part may be activated. It is governed by the parameter *τ*_*H*_, which can be defined per layer. By hallucination, the part $P_{i}^{n}$ is activated when the percentage of positive spectral components it covers exceeds the *τ*_*H*_:

$$\begin{array}{c} \frac{\left|\{k:k\in A_{C}^{\left(t\right)}\left(P_{i}^{n}\right)\wedge |X_{t}\left(k\right)|>0\}\right|}{|A_{C}^{\left(t\right)}\left(P_{i}^{n}\right)|}\geq \tau_{H}. \end{array}$$ (9)

If we set *τ*_*H*_ to 1, we obtain the default behavior (all of the covered spectral components must be present in the signal for parts to activate), while lowering of the parameter value leads to an increased number of activations across all layers.

By allowing activations in the presence of incomplete input, hallucination not only enables the model to fill-in the missing information, but also to yield the alternative explanations of the input signal. Namely, different parts of the model can explain the same fragments of information in the input. Hallucination boosts these alternative representations and enables the model to produce multiple explanations of the same input.

#### Inhibition

Inhibition performs the hypothesis refinement by reducing the number of part activations on individual layers. It provides a balancing factor in the model by reducing redundant activations, similar to lateral inhibition in the human auditory system [57]. Although the learning algorithm penalises parts redundantly covering the signal, some redundant parts are always present. During inference, each layer may therefore produce multiple redundant activations covering the same information in the input signal (hallucination also adds to the number of such activations).

The activation of the part $P_{i}^{n}$ is inhibited when different parts on the same layer cover the same spectral components in the input signal, but with a higher activation magnitude:

$$\begin{array}{c} \exists \left\{P_{j}^{n}..P_{k}^{n}\right\}:\wedge \left\{\begin{array}{c} \frac{|A_{C}^{\left(t\right)}\left(P_{i}^{n}\right)\setminus \cup \left\{A_{C}^{\left(t\right)}\left(P_{j}^{n}\right)..A_{C}^{\left(t\right)}\left(P_{k}^{n}\right)\right\}|}{|A_{C}^{\left(t\right)}\left(P_{i}^{n}\right)|}<\tau_{I}\\ \forall A_{M}^{\left(t\right)}\left(P_{j..k}^{n}\right)>A_{M}^{\left(t\right)}\left(P_{i}^{n}\right) \end{array}\right., \end{array}$$ (10)

where *τ*_*I*_ controls the amount of inhibition. Such control is needed, as complete inhibition of redundant parts’ activations is undesirable, due to the robustness the activations provide in a form of competing hypotheses about the information in the input signal. For example, a value of 0.5 will cause an activation to be inhibited if half of its coverage is already covered by stronger activations of other parts.

Alongside the hypothesis refinement, the removal of redundant activations also reduces noise in the input signal, which is usually manifested in a number of low-magnitude activations of parts on various layers. In combination with hallucination, inhibition provides an efficient way to control the explanatory power and robustness of the proposed model.

#### Automatic gain control

The model presented so far is time-independent. It operates on a time-frame-by-time-frame basis, where each time-frame in the time-frequency representation is processed independently from others. The automatic gain control mechanism (AGC) was introduced in the inference process in order to model short-time dependencies between frames. It operates on principles similar to automatic gain control contrast mechanism in human [58] and animal [59] perceptual systems. The mechanism allows linking of part activations through time by introducing time dependencies between activations.

**Fig 4:**
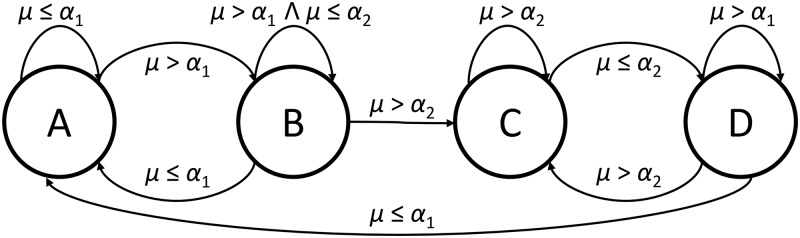
A finite state machine implementing the AGC mechanism. State A represents the normal behavior of a part, state B the boosting (onset), state C the sustain and state D the decay of the activation magnitude.

**Fig 5:**
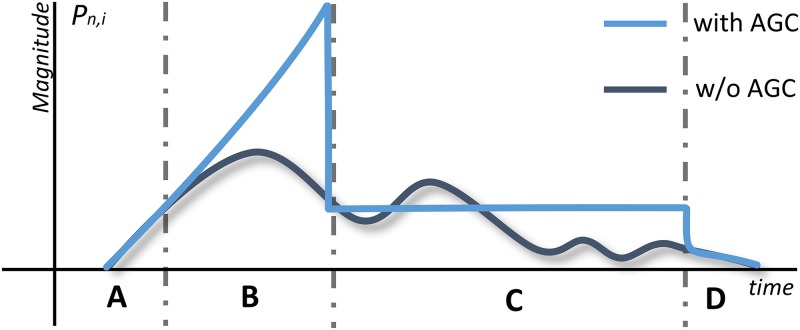
An abstract representation of AGC influence on part activations. Without AGC, activation magnitudes may notably fluctuate, especially towards the end of an event. AGC boosts the onset of an event and later keeps the activation magnitude on a fixed level until the offset.

The operation of the AGC is defined with a four-state finite state machine, as shown in Fig 4. AGC changes the activation of a part in the following manner: when the part is activated at a new location, and its activation persists, activation magnitude is initially boosted to accentuate the onset and later suppressed towards a stable value (see Fig 5).

The four AGC states represent: (A) normal part behavior, (B) onset, (C) sustain and (D) decay state. Transitions between the states are conditioned on the density of part activations *θ* within the time window *W*, which for the part $P_{i}^{n}$ at the time *t* is defined as:

$$\theta =\frac{1}{W}{\left\|\left[A_{M}^{\left(t-W+1\right)}\left(P_{i}^{n}\right),\ldots ,A_{M}^{\left(t\right)}\left(P_{i}^{n}\right)\right]\right\|}_{0}.$$ (11)

*α*_1_ and *α*_2_ are thresholds that control transitions between the states. The magnitude of a part activation for the individual states is calculated as:

$$\begin{array}{c} A_{M}^{\left(t\right)}\left(P_{i}^{n}\right)=\left\{\begin{array}{cc} A_{M}^{\left(t\right)}\left(P_{i}^{n}\right): & A,D\\ \sum_{f=t-W+1}^{t}A_{M}^{\left(f\right)}\left(P_{i}^{n}\right): & B\\ \tau_{S}: & C \end{array}\right., \end{array}$$ (12)

where *τ*_*S*_ represents a constant activation magnitude in the sustain state.

The mechanism operates on all layers; it has a short-term effect on lower layers and longer-term effect on higher layers (the window size *W* increases for each consecutive layer) in line with the complexity of concepts represented on different layers. The mechanism’s effect on the activation magnitude is shown in Fig 5. AGC stabilizes activations, boosts event onsets and produces an overall smoother model output with less fluctuation.

## Evaluation

Our proposed model is applicable to different MIR tasks in the audio domain, as presented in [45], as well as in the symbolic domain [46]. In this section, we demonstrate its usefulness for the multiple fundamental frequency estimation (MFFE), where the goal is to estimate which fundamental frequencies are present in the signal at individual time-frames.

### Choice of parameters

**Fig 6:**
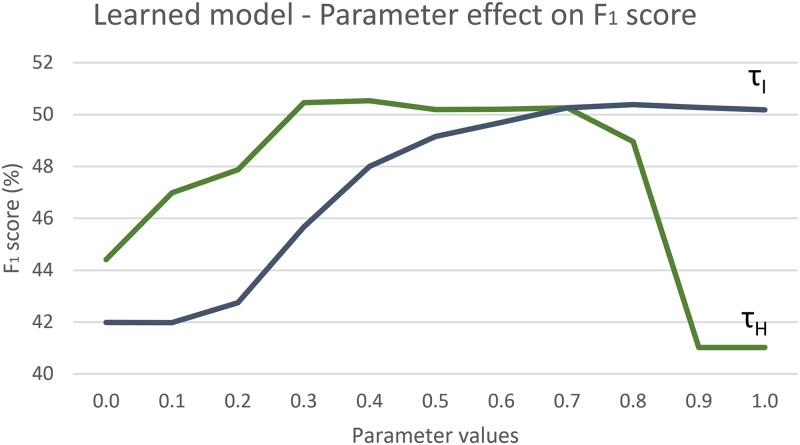
Piano transcription performance on the *AkPnBcht* folder from the MAPS dataset with different *τ*_*H*_ and *τ*_*I*_ values. The *x* axis represents parameter values, the *y* axis the *F*_1_ score.

Our model has several parameters, which influence learning and inference. We first evaluated the sensitivity of the proposed model to different values of its two most significant parameters: *τ*_*H*_ and *τ*_*I*_. Results in Fig 6 show that the model performance for MFFE is mostly stable, apart from extreme values. If *τ*_*H*_ that controls hallucination is set to a low value, the amount of activations increases drastically, as parts are allowed to hallucinate almost freely and vice-versa. High values produce few activations, so in both cases performance suffers. Similarly, a low value of *τ*_*I*_ (inhibition) results in a large number of part activations and subsequently worse performance.

Other parameters also have well defined roles and effects. The model is invariant to changes of *τ*_*P*_ above approximately 0.75 due to limitations imposed by *τ*_*C*_. High values of the latter result in small part candidate sets and insufficient coverage of the signal. AGC parameters *α*_1_ and *α*_2_ influence the stability of activations over time and only affect the performance if set to extreme values.

Because the model is not very sensitive to values of its parameters, we did not tune them specifically for each experiment, but chose to set them to common-sense values and keep them constant for all experiments. The input layer $L_{0}$ was based on a constant-Q transform with 345 frequency bins between 55 and 8000 Hz (48 per octave), a step size of 10 ms and a maximal window size of 100 ms. Training and the inference parameters *τ*_*H*_, *τ*_*I*_, *τ*_*P*_ and *τ*_*C*_ were set to values 0.7, 0.5, 0.9 and 0.005 respectively and AGC parameters to *α*_1_ = 0.2 and *α*_2_ = 0.5.

### Experiment

To evaluate the model for the multiple fundamental frequency estimation, we trained three layers of compositions on top of $L_{0}$, as described in the previous Section. A four-layer structure was sufficient for the model to learn a robust representation of pitch, as shown in our results.

Training is performed in an unsupervised manner on a training dataset. To assess how different training datasets influence the structure of the model, we trained the model on several large and small datasets: three small datasets consisting of individual isolated instrument sounds (piano, flute and guitar), two medium-sized datasets of popular music (the Beatles and Queen albums) and a large dataset of polyphonic piano music. A comparison of the learned structures showed that the size of the learned models did not vary significantly. All models contained a small number of compositions on all layers (in total between 50–60), with very similar structures. The average Jaccard index per layer was 0.586 and 0.381 for $L_{1}$ and $L_{2}$ respectively. It was higher for models trained only on individual instrument samples (0.764 and 0.56) or only polyphonic music (0.778 and 0.522). Only identical parts were counted when calculating the index, although other parts were also similar (e.g. compositions that have three out of four subparts and offsets in common). Such small size and similarity of the learned models is the consequence of two features of the proposed model: relativity and shareability, which enable learning of generalized concepts, such as pitch, encoded in small models, which can be trained on small datasets.

We therefore decided to perform all our experiments on a model trained on individual Bösendorfer model 225 (From the EastWest Ultimate Piano Collection) piano notes (these were not included in the testing datasets), which makes training fast, but still yields good results. The learned model contained only 23, 12 and 16 parts on layers 1, 2 and 3 respectively. The amount of parts on $L_{3}$ layer did not exceed those on $L_{1}$ and $L_{2}$, as could be expected in compositional models, because regularization during the learning process balances the coverage and the amount of generated parts per layer.

To use the model for MFFE, we exploited its transparency, which enabled us to interpret the activations of parts on the layer $L_{3}$ and directly map them onto the frequency axis, thus extracting a set of fundamental frequencies at each time-frame. No additional supervised machine learning models were therefore used for estimation of fundamental frequencies.

To assess the robustness of the learned pitch concepts, we tested the model for MFFE on four distinct datasets: MAPS M [60], containing piano-synthesized MIDI files, MAPS D, containing recordings of the Disklavier [60], Su & Yang dataset [61], containing mixtures of piano and string instruments, and a dataset of folk songs sung by choirs of 2–4 singers (available at osf.io/f7h3r).

For all datasets, we compared our results to three other methods: DNMF decomposition of the time-frequency representation [38], where DNMF was trained on 70% of the dataset and tested on the remaining 30%, the Klapuri’s multiple F0 estimation method [29] and two approaches presented by Benetos and Weyde [27, 62]. For the Klapuri’s method, we used 30% of the annotated dataset to fine-tune the salience threshold parameter.

Results are given in Table 1. They show that the proposed model learns a robust representation of pitch and has good generalisation power, as it yields consistent results on different datasets. While other approaches, such as DNMF or Benetos’, achieve better scores on some datasets, they overfit the timbres they were trained on (e.g. DNMF was trained on the majority of the MAPS dataset), so their performances in cases where timbre is not so well defined (e.g. the folk song dataset containing choir singing) are poor.

**Table 1:** Comparison of CHM, DNMF, Klapuri and Benetos approaches. *F*_1_ scores in %, running times and memory usage for 1 minute of audio for different datasets and different transcription methods are shown. *F*_1_ scores are frame-based scores calculated in accordance to MIREX MFFE evaluations [63].

Dataset	CHM	DNMF	Klapuri [29]	Benetos [27]	Benetos [62]
MAPS MIDI	52.6	**61.6**	56.0	56.7	56.7
MAPS D	51.8	57.1	52.5	50.1	**62.6**
Su & Yang	**48.9**	32.6	48.0	40.3	55.6
Folk song	**49.3**	35.0	31.8	27.5	16.2
Average *F*_1_	**50.7**	46.6	47.1	43.7	47.8
Running time (s)	6.2	5.7	19.4	188.1	87
RAM Usage (MB)	63.8	120.0	43.2	1914.2	716.5

Although trained only on piano notes, the proposed model unsupervisedly learned the concept of pitch in a robust manner, without (over)fitting to specific templates of a single instrument. It is the most accurate of all compared approaches on the folk song dataset, where it demonstrates its robustness. A singing transcription is difficult for most algorithms based on harmonic templates (which include all compared algorithms), as the vocal timbre changes not only between songs (different performers), but also within a song (different vowels, stress etc.). It is therefore difficult to capture the timbre with a template, which results in poor transcription performance, especially in terms of precision. In addition, these songs originate from field recordings of folk music that are performed by amateur singers and recorded in everyday environments with portable audio equipment. Thus, they significantly differ from the studio-level or synthesised recordings. The CHM, with its multilayer representation, hallucination, inhibition and AGC mechanisms, achieves performance comparable to other datasets, while the compared methods perform significantly worse (Kruskal-Wallis test *χ*^2^ = 56.8, *p* < 10^−11^).

### Error analysis

We analysed the model’s output with respect to the manually annotated ground truth to assess the most typical errors made by the proposed model. Four types of errors are frequent: offset localisation, semitone errors, harmonic (octave) errors and pitch fluctuation.

**Fig 7:**
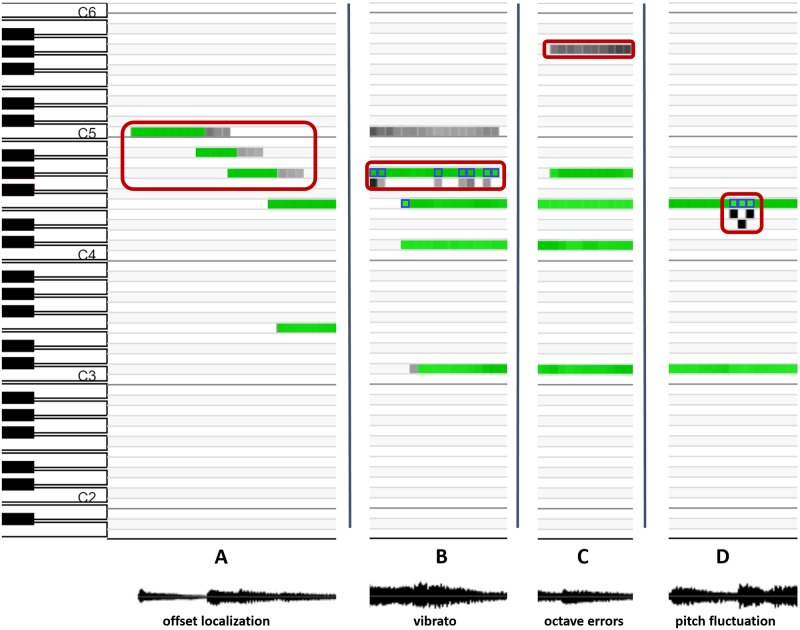
The most frequent errors produced by the CHM. Ground truth annotations are displayed in green, the CHM activations are shown in grey. Activations that are not aligned with the ground truth represent false positive errors. Additionally, false negatives are outlined with blue color.

Offset localisation errors frequently appear in recordings with strong reverberation, where an event is prolonged and is detected after the instrument stopped playing. The AGC mechanism may additionally prolong the detected offsets, so the combination of both factors reflects in longer durations of identified events, as shown in Fig 7-A. The singer’s vibrato can cause the detected pitch to shift up or down in individual frames, which may cause semitone errors, as the groundtruth usually reflects the desired and not the actual pitch within a time-frame (Fig 7-B). Octave and other harmonically related errors are a common source of errors for most algorithms due to sharing of harmonics between harmonically related tones. CHM is no exception, especially in recordings where instruments contain many strong harmonic components (Fig 7-C). Voice fluctuations are commonly present in singing, especially when singers sing a capella (without a support of instruments). Pitch may fluctuate at onsets of syllables, resulting in the spread of energy over several semitones, similar to vibrato, which leads to pitch estimates that differ from the ground truth, as may be observed in Fig 7-D.

## Discussion

The proposed CHM model offers a novel approach to music information retrieval and analysis, specifically for the music transcription. It provides a compact hierarchical representation of the content of a music signal through activations of learned concepts over several layers. We demonstrated its effectiveness by using a model learned in a completely unsupervised manner for the multiple fundamental frequency estimation. This was possible due to the model’s transparency, where part activations can be interpreted meaningfully and projected to the input layer.

When compared to specialized approaches, the proposed algorithm may not perform as well as the current state of the art, which is expected, as it is not tuned for a specific task. For comparison, one of the best MAPS M transcription scores is 77.1%, reported by Weninger et al. [37]. His approach differs significantly from ours—it is based on a support vector machine classifier, which was trained on a large portion of the MAPS dataset (approx. 80% of the dataset).

The deep network approaches for MFFE [41–44] also typically use a large proportion of the dataset for training. Bock and Schedl [41] evaluated a recurrent neural network model on four piano music datasets, including MAPS MIDI and MAPS D. They reported a high *F*_1_ score (up to 93.5%) for note onset detection; however, they also used a significant amount of the datasets for training and validation (approximately 75% and 9.4% on average per dataset for training and validation respectively). Nam et al. [42] reported results for 30 second excerpts from the MAPS dataset (74.4% frame-level *F*_1_ score) by using roughly 60% of the dataset for training and 25% for validation.

The reason for this large proportions of training samples is that MFFE datasets are relatively small. This is due to the fact that annotations require expert knowledge and a significant amount of time. The annotations can thus not be crowdsourced, as for example in image labelling, where deep networks are very successful. It thus becomes necessary to include a significant amount of the available data into a training set, retaining only a small portion (down to 10% in several cases) for testing. Results are assumed to generalize over the whole dataset, and there is no information on how these models would perform on more diverse datasets, and for instruments with different timbres.

In comparison, our model was trained on only a small set of 88 piano key samples not present in the MAPS dataset. Although the CHM does not reach the accuracy of such tuned approaches, it is able to generalize and perform well in a variety of cases where the source is not so well defined, as shown in our evaluation on the Su & Yang and Folk song datasets. We may therefore conclude that the CHM extracts timbre-invariant features from the audio signal, which, combined with a robust inference mechanism, lead to a stable performance in various scenarios.

### Real-time performance

An added feature of the proposed approach lies in the small sizes of learned models, which are consequences of part relativity and shareability. The computational complexity of inference with such small models is low, so CHM can be used for transcription in real-time scenarios. Table 1 lists running times and memory consumption of all compared algorithms for one minute of audio measured on a system with 16GB RAM and an Intel Xeon E5520 2.26GHz processor using a single thread. The CHM and DNMF are the fastest, with approximately ten-to-one ratio of audio length over processing time, followed by Klapuri (approx. three-to-one ratio). Both approaches by Benetos and Weyde with 1.5-to-three and one-to-three ratio are not usable in real-time scenarios, as next to high running times, they also require the entire audio file for processing. The memory consumption of the proposed approach is also low—it uses approximately half the memory in comparison to DNMF, and around 50% more than Klapuri’s approach.

In addition, the approach is parallelizable, as parts on a layer can be inferred independently and thus in parallel. The speed, small memory consumption and robustness of our approach make it suitable for real-world use, and applicable within embedded systems and mobile devices with multiple cores and low processing power per core.

## Conclusion and future work

We introduced a compositional hierarchical model for music information retrieval and music analysis. We showed how the model is used for music transcription and evaluated its ability to perform in real-time. This ability enables the usage of the model for a number of real-world applications and platforms, such as embedded and mobile systems. The model is constructed by unsupervised learning on a set of audio recordings and contains compositions of parts reflecting the statistical regularities in the learning set, encoding simple concepts on lower layers and complex concepts on higher layers. The model’s transparency enables insights into the model’s structure and consequently into the music concepts represented by individual parts, such as pitch partials, pitches and harmonies. The relativity and shareability of parts enable a compact representation of the learned concepts, while robustness is achieved by incorporating inhibition, hallucination and AGC mechanisms, as presented in the *Inference* Section.

### A different deep architecture

The compositional hierarchical model shares some similarities with deep learning architectures. It is similar in terms of learning a variety of signal abstractions on several layers of granularity. The learning procedure is similar: the structure is built layer-by-layer. However, unlike most deep architectures, CHM is learned in an entirely unsupervised manner, so no annotated dataset is needed for training and validation. In addition, several aspects of the model set it apart from other architectures.

Transparency is manifested in the compositional nature of the model. Parts are compositions of subparts and their activations are directly observable and interpretable (each activation can be projected to the input layer and its effect observed). In contrast, most other neural-network-based deep architectures offer no clear explanation of the underlying feature extraction process and the meaning of the extracted features, with the exception of convolutional neural networks, which partially and indirectly offer explanations of their nodes [64]. Transparency enables the model to be used directly as a classifier by observing and interpreting part activations, as we show in our evaluation task.

In addition, the hallucination and the inhibition mechanisms facilitate the production of alternative explanations of the input during inference. By suppressing the most prominent explanation, the model can produce alternative hypotheses previously suppressed by this explanation. Combined with transparency, this makes the model a suitable music analysis tool, where signal contents can be visualized and interpreted as activations of high-to-low level concepts encoded by the model, which may also be interactively manipulated to obtain alternative hypotheses.

Relativity and shareability of parts enable efficient encoding of the learned concepts and lead to a small number of parts needed to represent complex concepts. A part in the proposed model is defined by the relative distance between its subparts and can be activated on different locations along the frequency axis. Therefore, the large amount of layer units that, for example, convolutional networks need to cover the entire range of frequencies, is not necessary. Relativity is accompanied by part shareability: parts on a layer may be shared by many compositions on higher layers. Although this feature is similar to other deep representations, relativity takes shareability a step further: a set of subparts may form several new relative compositions on a higher layer representing different entities and may thus be efficiently reused. The learned models therefore contain a small number of parts, which also enables the use of small datasets for training a small number of trainable parameters, which lead to a very fast inference. This is also evident in the presented evaluation, where a small set of samples was used to train a three-layer model that performed well on several different datasets.

### Future work

The model is general and can be used for audio-based as well as symbolic MIR tasks, including automated chord estimation and mood estimation [45], symbolic pattern discovery [46] and multiple fundamental frequency estimation presented in this paper. For the latter, the model serves both as a feature extractor, as well as a classifier. We also demonstrated the model’s robustness to varying timbres and audio signals which were recorded in suboptimal conditions.

The proposed approach is naturally expandable to the time domain, which we already demonstrated by its application to the symbolic pattern discovery task [46]. In our future work, we aim to further develop the model as a general purpose model for music information retrieval and music analysis. Future work includes stacking of models applied to the audio and symbolic domains, thus introducing a single model which covers different MIR tasks and is suitable for real-world application. We intend to extend the model to encode long-term temporal dependencies of music events, thus encoding concepts such as melodic lines, chord progressions and rhythmic patterns. Such a unified framework which models the spatial (frequency) and temporal structure of music events should improve performance for a variety of MIR tasks and potentially eliminate the need for additional temporal processing stages, such as the hidden Markov models.
